# Clinical Observations—Bismuth in Cholera Infantum

**Published:** 1894-04

**Authors:** Adolph Lippe


### CLINICAL OBSERVATIONS—BISMUTH IN CHOLERA INFANTUM.

ADOLPH LIPPE, M. D.

A child, six months old, fed with scalded cream from the bottle, and extraordinarily fleshy, was taken sick with cholera infantum at M——, Pa. The resident (self-styled homœopathic) physician was sent for, who administered some "*odorate*" medicine, and in addition applied a spice plaster to the abdomen.

The child evidently growing worse, the spice plaster was changed to a mustard plaster, and the internal medicine made still more "*odorate.*" The child still growing worse, a *fly-blister* was resorted to, and the internally administered drug made still more "*odorate.*" The tender mother, remonstrating against such *homœopathic* treatment, was told that he (the doctor) belonged to a much more liberal school than did the old friend of the mother. The poor child grew much worse under the bogus treatment, and the distracted mother brought it to the city. It was found continuously restless; crying all the time; diarrhœa, watery and very offensive, worse at night; continuous, unquenchable thirst for *cold* water; had taken no nourishment for some days; head hot (not the blistered abdomen); had passed no urine for a long time, and this discharge had gradually grown less in quantity. One dose of Arsenicum$^{40m}$ was followed by a very quiet night, and the poor child was much better for forty-eight hours, when the diarrhœa returned, although less violently; the thirst had ceased entirely, but the appetite did not return; the child now began to vomit, *but only* the water it had taken, and this even in the smallest quantities, and at once; it cried more at night, though not violently, whining more from discomfort than from acute pain—probably nausea. The child rejected all nourishment, nothing would tempt it; the stools were thin and offensive, but not very frequent; it rolled its head at times, especially when crying. *Silicea* had caused no permanently good results. One dose of *Bismuth*$^{2c}$ (Lehrmann), of which the curative effects have been confirmed—only water thrown up, while other substances entering the stomach are retained—changed the whole aspect of the case, and forty-eight hours after the administration of this single dose the child was convalescent; slept all night; the pale cheeks resumed their former color; it took its former accustomed nourishment, and required no further treatment. The case confirms positively all the well-known principles of Homœopathy, and shows the folly of the so-called *liberal* practice.—*Hahn. Monthly, Vol. IV, Sept.*, 1868.

